# Intramuscular Pseudoaneurysm Following Minimally Invasive Pedicle Screw Fixation for Lumbar Fracture Treated With Thrombin Injection: A Report of a Rare Case

**DOI:** 10.7759/cureus.93239

**Published:** 2025-09-25

**Authors:** Bashar Altunbi, Oludare Ashaolu, Moe Thidar Aung

**Affiliations:** 1 Spinal Surgery, Swansea Bay University Health Board, Swansea, GBR; 2 Trauma and Orthopedics, Swansea Bay University Health Board, Swansea, GBR; 3 Internal Medicine, The Queen Elizabeth Hospital, Central Adelaide Local Health Network, Adelaide, AUS

**Keywords:** case report, minimally invasive spine surgery, pedicle screw, pseudoaneurysm, vascular complication

## Abstract

Minimally invasive surgery (MIS) techniques offer reduced tissue disruption and faster recovery, but rare vascular complications such as pseudoaneurysm may still occur. We present the case of a 66-year-old female patient with ankylosing spondylitis who sustained an unstable L2 fracture. Following navigated MIS pedicle screw fixation, she developed delayed paraspinal swelling. Initial CT demonstrated a paraspinal hematoma, and subsequent CT angiography confirmed an intramuscular pseudoaneurysm, which was successfully treated with fluoroscopy-guided thrombin injection. The patient remained asymptomatic with no recurrence at six-week and five-month follow‑up. This case underscores the importance of maintaining a high index of suspicion for vascular injury in postoperative patients with ankylosing spondylitis and demonstrates the effectiveness of fluoroscopy‑guided thrombin injection as a minimally invasive treatment option, contextualized by a brief literature review of diagnostic challenges and evolving management strategies.

## Introduction

Minimally invasive surgery (MIS) techniques offer advantages such as reduced tissue disruption, shorter hospital stays, and faster recovery. However, vascular complications, though rare, can occur. Pseudoaneurysms result from transmural arterial rupture, forming a hematoma that communicates with the arterial lumen. Their incidence following spinal instrumentation is low but potentially life-threatening [1,2].

Patients with ankylosing spondylitis present additional challenges due to altered spinal biomechanics and frequent osteoporosis, which may predispose them to unstable fractures and surgical complications, including vascular injury. This report describes a rare case of pseudoaneurysm after MIS pedicle screw fixation for lumbar fracture in a patient with ankylosing spondylitis, emphasizing the need to consider vascular injury in patients with delayed postoperative swelling.

## Case presentation

**Figure 1:**
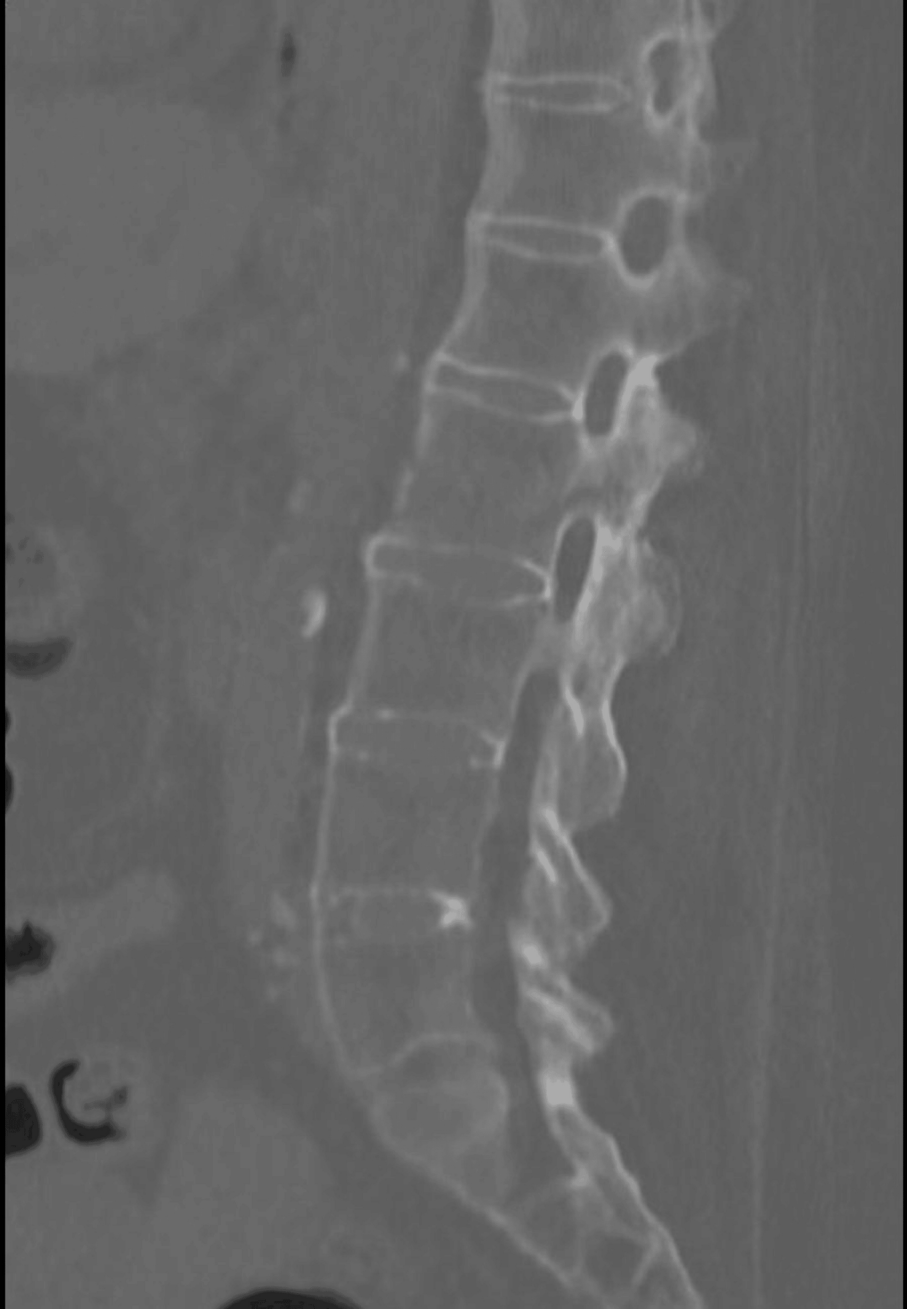
Preoperative CT of L2 fracture Sagittal CT image showing an unstable L2 chalk-stick fracture prior to surgical intervention.

A 66-year-old female patient with ankylosing spondylitis sustained a fall while cooking, resulting in a superficial burn and an unstable L2 chalk-stick fracture typical of ankylosing spondylitis. Figure 1 shows the preoperative CT demonstrating the unstable L2 fracture.

**Figure 2:**
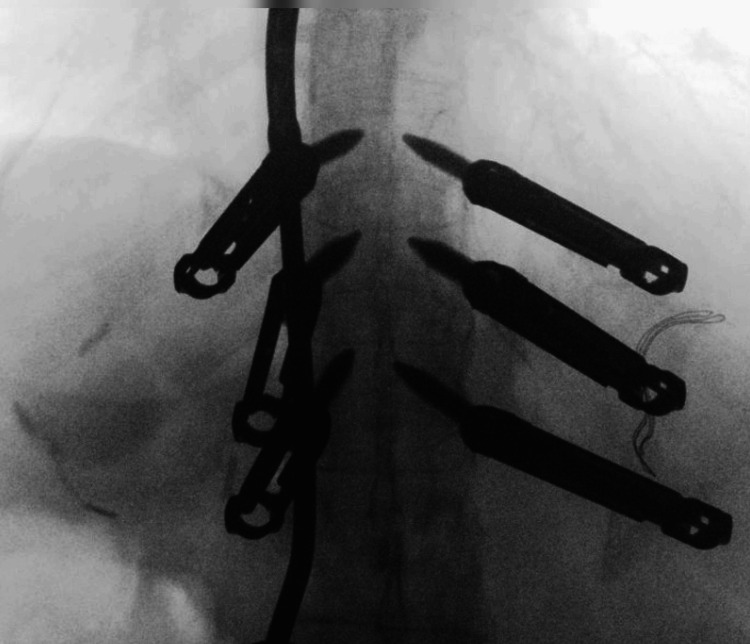
Intraoperative imaging of pedicle screw fixation Fluoroscopic image showing intraoperative pedicle screw placement from T11 to L1 above the L2 fracture site. The lower instrumentation from L3 to L5 was performed subsequently but is not included in this image, as the vascular complication was localized to the upper construct.

Upon admission, she was found to be in new-onset atrial fibrillation. Following multidisciplinary optimization, she underwent navigated MIS posterior stabilization using percutaneous pedicle screws from T11 to L1 and L3 to L5, sparing L2. Intraoperative fluoroscopy confirmed accurate pedicle screw placement from T11 to L1 above the fracture site, as illustrated in Figure 2. The lower instrumentation was performed subsequently and is not included in this image.

**Figure 3:**
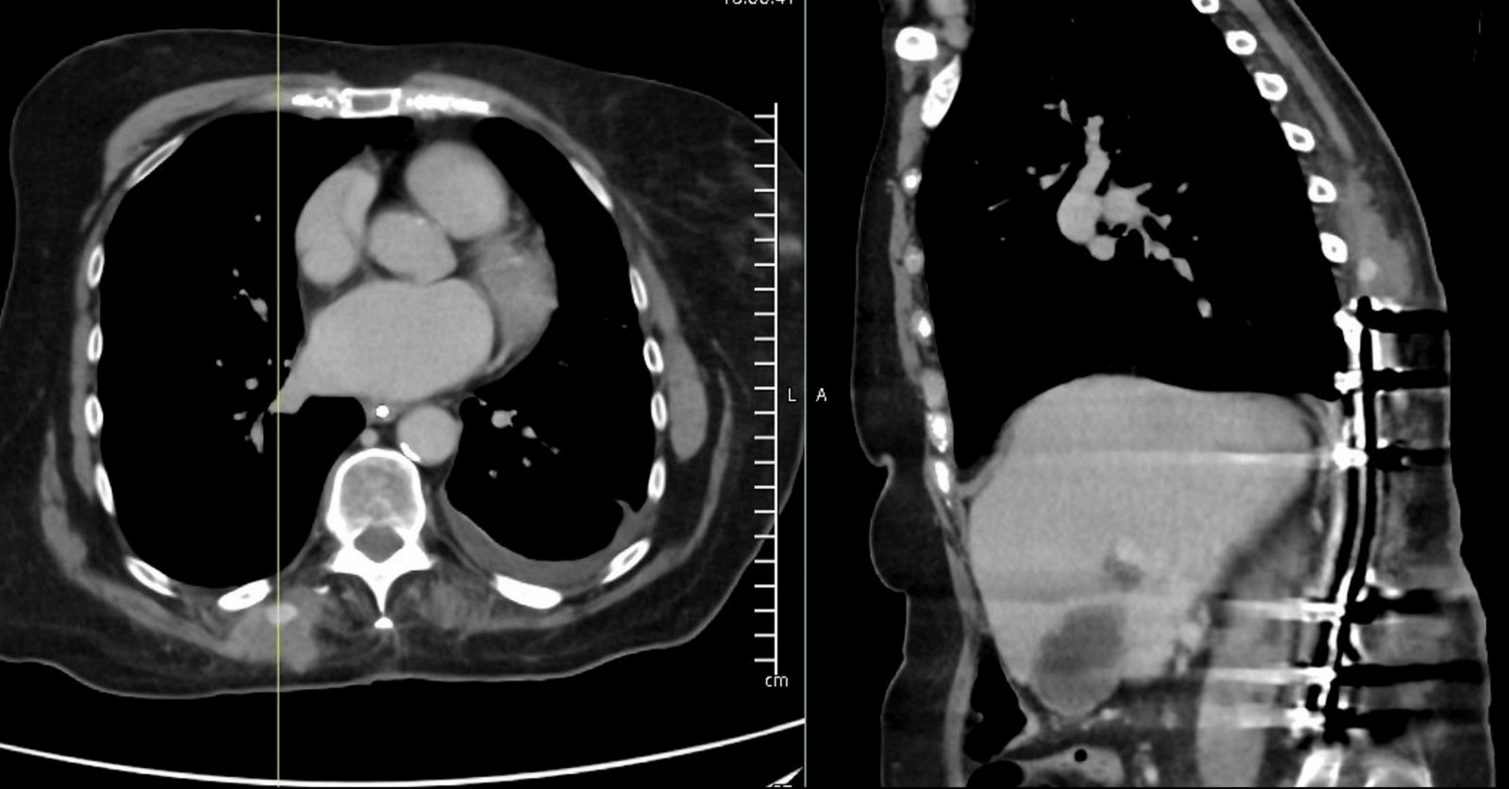
CT angiography demonstrating pseudoaneurysm Axial (left) and sagittal (right) CT angiography images showing a well‑defined intramuscular hematoma adjacent to the pedicle screw tract. The axial image includes a reference line indicating the corresponding sagittal level. Imaging features are consistent with a pseudoaneurysm.

The operation was uneventful, though High Dependency Unit (HDU) monitoring was required postoperatively for cardiologic instability. Recovery remained uncomplicated until postoperative day 12, when the patient developed sudden, painful swelling in the right paraspinal region. An initial CT scan revealed a moderate paraspinal hematoma near the screw site. A subsequent CT angiography confirmed active arterial bleeding from an intramuscular vessel, forming a pseudoaneurysm, as illustrated in Figure 3.

Emergency fluoroscopy-guided thrombin injection was performed by interventional radiology with complete resolution of symptoms within 48 hours. The patient had been commenced on apixaban 48 hours after surgery for newly diagnosed atrial fibrillation and was receiving anticoagulation at the time of presentation. She was discharged in stable condition after several days of inpatient monitoring, and remained asymptomatic with no recurrence at six‑week and five-month clinic follow‑up.

## Discussion

Pseudoaneurysms result from arterial wall disruption, with blood confined by surrounding tissue. If untreated, they can enlarge, rupture, or compress adjacent structures, leading to significant morbidity [1,2]. Although uncommon after spinal instrumentation, pseudoaneurysms have been reported, typically linked to direct trauma during pedicle screw placement. MIS approaches, while advantageous for recovery, provide limited visualization compared with open surgery, where wider exposure often allows earlier recognition and control of bleeding. This difference underscores the need for vigilance after MIS fixation, particularly in patients with additional risk factors.

Diagnosis is often challenging because pseudoaneurysms may initially present as localized swelling or hematoma, mimicking common postoperative changes. Cross-sectional imaging, particularly CT angiography, plays a crucial role in distinguishing pseudoaneurysm from other postoperative complications [3,4]. While Doppler ultrasound can serve as a screening tool in some settings, particularly in resource-limited settings, CT angiography was prioritized in this case due to its rapid availability, high diagnostic accuracy, and ability to guide urgent intervention. Early recognition is critical, as delayed diagnosis may result in rupture or persistent bleeding.

Management strategies have evolved from open surgical repair to minimally invasive interventional radiology. Image-guided thrombin injection and coil embolization are effective and less invasive than open surgery, with high success rates [1,4,5]. In selected stable patients, conservative management may also be appropriate [6]. Our case reinforces the role of interventional radiology in providing rapid, definitive treatment with minimal morbidity. The patient remained asymptomatic, and no recurrence was observed during follow‑up, confirming the durability of thrombin injection in this context. Although ankylosing spondylitis is not directly associated with vascular malformations, its characteristic rigid spinal anatomy and frequent co‑existing osteoporosis predispose patients to unstable fractures and technically demanding surgery. These factors may increase the likelihood of iatrogenic vascular injury during instrumentation or fracture management, particularly in the context of minimally invasive approaches where visualization is limited. The patient was receiving apixaban at the time of presentation, which may have contributed to hematoma expansion, although the primary mechanism was iatrogenic vascular injury.

A review of the literature highlights that lumbar artery pseudoaneurysms are the most frequently reported vascular injuries following spinal or traumatic interventions [3,5-7]. This case is distinct in describing an intramuscular pseudoaneurysm after MIS pedicle screw fixation in a patient with ankylosing spondylitis, successfully treated with fluoroscopy‑guided thrombin injection - a modality rarely reported in this context. A comparative summary of previously published cases, along with our present case, is provided in Table 1, highlighting vessel involvement, mechanism of injury, time to diagnosis, treatment modalities, and clinical outcomes.

**Table 1:** Summary of reported cases of vascular injury in spinal surgery, including the present case This table summarizes key published cases of vascular injury associated with spinal procedures, as well as the present case, including vessel involvement, mechanism, time to diagnosis, treatment, and outcomes. MIS: minimally invasive surgery

Author/publication year	Vessel involved	Mechanism of injury	Time to diagnosis	Treatment modality	Clinical outcome
Sugimoto et al. (2013) [3]	Lumbar artery	Pedicle screw insertion	Intraoperative	Coil embolization	Complete resolution
Vashisht et al. (2019) [6]	Lumbar artery	Blunt trauma	Delayed (days post‑injury)	Conservative management	Complete resolution
Karaikovic et al. (2010) [4]	Lumbar artery	Intradiscal surgical trauma	Intraoperative	Coil embolization	Complete resolution
Mohan et al. (2017) [5]	Multiple vessels	Traumatic pseudoaneurysm	Variable (2 hours – 75 days)	Endovascular embolization	Good long-term outcome
Tulsyan et al. (2007) [7]	Visceral arteries	Various etiologies	Not specified	Coils and stent grafts	Successful long‑term outcomes
Present case (2025)	Intramuscular branch vessel	MIS pedicle screw fixation	Postoperative day 12	Thrombin injection	Complete resolution

## Conclusions

Iatrogenic pseudoaneurysm is a rare but important complication following MIS pedicle screw fixation. Clinicians should maintain a high index of suspicion for vascular injury when encountering delayed pain and swelling in postoperative patients. Prompt imaging and minimally invasive interventional therapy, such as thrombin injection, are critical for achieving favorable and durable outcomes, as demonstrated in our case with recurrence‑free follow‑up at six weeks and five months.
